# Hypomethylation and downregulation of miR-23b-3p are associated with upregulated PLAU: a diagnostic and prognostic biomarker in head and neck squamous cell carcinoma

**DOI:** 10.1186/s12935-021-02251-w

**Published:** 2021-10-26

**Authors:** Zirong Huo, Xiaoguang Li, Jieyu Zhou, Yuqin Fan, Zhentao Wang, Zhihua Zhang

**Affiliations:** 1 Department of Otorhinolaryngology Head and Neck Surgery, Shanghai Ninth People’s Hospital, Shanghai Jiaotong University School of Medicine, Shanghai, China; 2 Ear Institute Shanghai Jiaotong University, Shanghai, China; 3 Shanghai Key Laboratory of Translational Medicine on Ear and Nose Diseases, Shanghai, China

**Keywords:** HNSCC, Bioinformatics, PLAU, Prognosis, Methylation, miRNA

## Abstract

**Background:**

DNA methylation and miRNA-target genes play an important part in the early development of various tumors and have been studied as tumor biomarkers. Although previous studies have reported a cluster of molecular events (such as aberrant alterations of genomics and epigenetics), little is known of the potential biomarkers for early diagnosis and prognostic evaluation in head and neck squamous cell carcinoma (HNSCC).

**Methods:**

Multiple bioinformatics tools based on The Cancer Genome Atlas (TCGA) database and clinical samples were applied to evaluate the beneficial biomarkers in HNSCC. We focused on the role of plasminogen activator urokinase (PLAU), including diagnostic and prognostic significance, gene expression analysis, aberrant DNA methylation characteristics, interaction of miRNAs and associated signaling pathways.

**Results:**

We found that PLAU was aberrantly upregulated in HNSCC, regardless of the mRNA or protein level. The results of receiver operating characteristic (ROC) curve and Cox regression analysis revealed that PLAU was a diagnostic and independent prognostic factor for patients with HNSCC. Hypomethylation of PLAU was closely related to poor survival in HNSCC. Additionally, miR-23b-3p was predicted to target PLAU and was significantly downregulated in HNSCC tissues. Therefore, our findings suggested that PLAU functioned as a promoter in the pathological process of HNSCC. DNA hypomethylation and downregulation of miR-23b-3p were associated with PLAU overexpression. Finally, our findings provided evidence of a significant interaction between PLAU-target and miRNAs-target pathways, indicating that miR-23b-3p suppresses malignant properties of HNSCC by targeting PLAU via Ras/MAPK and Akt/mTOR signaling pathways.

**Conclusions:**

PLAU is overexpressed and may serve as an independent diagnostic and prognostic biomarker in HNSCC. Hypomethylation and downregulation of miR-23b-3p might account for the oncogenic role of PLAU in HNSCC.

**Supplementary Information:**

The online version contains supplementary material available at 10.1186/s12935-021-02251-w.

## Background

Head and neck squamous cell carcinoma (HNSCC) is the sixth most common malignancy worldwide [1]. More than 800,000 patients are diagnosed and more than 400,000 patients die of HNSCC per year [2]. HNSCC comprises a heterogeneous group of tumors that arise from the squamous epithelium of the oral cavity, oropharynx, larynx and hypopharynx, and is characterized by low survival rates, high recurrence rates, and/or prominent lymph node involvement [3]. Although the resent decades have seen many advancements in treatments, including combinations of surgery, radiotherapy and chemotherapy, the overall survival rate of patients with HNSCC has not markedly increased [4]. For patients with HNSCC, prognosis prediction is crucial to be considered suitable for personalized treatment by physicians. Although clinical parameters such as the primary tumor, regional lymph nodes, and metastasis (TNM) classification are applied to predict the outcome and make therapeutic decisions, they have a low accuracy of prediction in many situations [5, 6]. Therefore, it is urgent to identify novel prognostic biomarkers to improve survival for patients with HNSCC.

Growing evidence has shown that malignant tumors are genetic and epigenetic diseases. Epigenetic changes play an important role in oncogenesis and progression of tumors and might occur before genetic variation [7]. As a stable epigenetic modification, DNA methylation contributes to the spatiotemporal regulation of gene expression [8]. The hypomethylation of the entire gene sequence is the earliest epigenetic change, while hypermethylation appears to be associated with transcriptional repression, an important mechanism of tumorigenesis [9]. Plasminogen activator urokinase (PLAU), also known as plasminogen activator, is a serine protease that converts plasminogen to plasmin [10], and can hydrolyze extracellular matrix remodeling proteins and activate growth factors [11]. There is growing evidence that PLAU plays a vital role in the genesis and development of various cancers, including colorectal cancer, breast cancer, and esophageal cancer [12–14]. However, limited studies have examined PLAU in HNSCC, and consequently, its role remains largely unknown.

In this study, we evaluated the expression and prognosis of PLAU in HNSCC. Aberrant methylation and miRNA-target regulation were assessed to identify PLAU upregulation in HNSCC. Additionally, diagnostic capability was evaluated using the receiver operating characteristic (ROC) curve. The investigation of PLAU based on multiple bioinformatic tools provides us with a strong evidence that it may be considered as an independent prognostic indicator for patients with HNSCC.

## Methods

### Differentially expressed gene analysis

Two datasets of gene expression profiles, including GSE55550 and GSE40290, were selected from the Gene Expression Omnibus (GEO, https://www.ncbi.nlm.nih.gov/) dataset [15]. The database from The Cancer Genome Atlas (TCGA, https://portal.gdc.cancer.gov/) was also included in our study. The Limma package (version: 3.40.2) of R software was used to detect the differentially expressed genes (DEGs) in each profile.

### Expression analysis of PLAU

The TCGA database was used to investigate mRNA expression level of PLAU, while a comprehensive evaluation on the basis of The Human Protein Atlas (THPA, https://www.proteinatlas.org/) was applied to assess the protein expression level of PLAU. Moreover, the Wilcoxon rank-sum test was employed to explore the correlation between PLAU expression and clinical characteristics of patients with HNSCC from TCGA database, including age, sex, smoking habit, tumor stage, lymph node metastasis, distant metastasis, and clinical stage.

Twenty patients who were diagnosed with HNSCC were retrospectively recruited in Shanghai Ninth People’s Hospital Affiliated to Shanghai Jiaotong University School of Medicine from January 2021 to March 2021. Twenty pairs of samples were classified into two groups: carcinoma and para-carcinoma. RNA was extracted and reverse-transcribed to cDNA. Quantitative polymerase chain reaction (qPCR) was applied to evaluate PLAU expression at the RNA level. The primers used were as follows: PLAU: (forward) TCGCTCAAGGCTTAACTCCAACAC, (reverse) ACGGATCTTCAGCAAGGCAATGTC; GAPDH: (forward) TCAAGAAGGTGGTGAAGCAG, (reverse) CGTCAAAGGTGGAGGAGTG. Immunohistochemistry (IHC) was performed as previously described [16]. The issue sections were incubated with anti-PLAU antibody (1:300, ab133563; Abcam, Shanghai, China) overnight at 4 °C.

### Prognostic evaluation of PLAU

Raw counts of RNA-sequencing data and related clinical information of HNSCC were downloaded from the TCGA dataset. Kaplan–Meier (KM) survival analysis was performed to reveal the correlation between gene expression and 5-year overall survival (OS). Multivariate Cox regression analysis was performed to further explore whether PLAU expression was an independent factor out of the other clinical variables for prognostic evaluation of an individual with HNSCC.

### Methylation analysis of PLAU

We detected the methylation status of PLAU using UALAN (http://ualcan.path.uab.edu/) [17]. We additionally evaluated the association between the PLAU methylation level and clinical characteristics including age, sex, and stage. Additionally, MethSurv (https://biit.cs.ut.ee/methsurv/) [18] was performed to detect the correlation between the prognosis of patients with HNSCC and the position distributions of methylation around CpG islands. We also investigated the correlation between three DNA methyltransferases (DNMT1, DNMT3A, and DNMT3B) and PLAU on the basis of TCGA database.

### Pathways and protein–protein interaction analysis

The signaling pathway involved in HNSCC was predicted by GSCALite (http://bioinfo.life.hust.edu.cn/web/GSCALite/) [19]. Additionally, the critical molecules among the activated pathways were determined, and the correlation between the expression levels of PLAU and the molecules was detected by Spearman’s correlation analysis. The protein–protein interaction (PPI) network from STRING (https://string-db.org/) [20] was depicted and visualized by Cytoscape software v3.8.2 [21].

### Analysis of PLAU-targeted miRNAs

The PLAU-targeted miRNAs were predicted by TargetScan, miRDB and miCODE, and the overlapping miRNAs were further filtered. Next, we assessed the expression levels of different miRNAs based on TGFA database. MiRNA-dependent Kyoto Encyclopedia of Genes and Genomes (KEGG) pathway and Gene Ontology (GO) analysis were additionally performed via DIANA-miRPath v3.0 (http://www.microrna.gr/miRPathv3) [22], an online software suite dedicated to the assessment of miRNA regulatory roles and the identification of controlled pathways.

### Diagnostic analysis of PLAU

The diagnostic ability of PLAU in HNSCC was evaluated using the ROC curve. To further compare the diagnostic capability between PLAU and other common biomarkers in malignant tumors, epidermal growth factor receptor (EGFR) was applied to react the difference.

### Statistical analysis

All data were analyzed by the SPSS statistical package (version 24.0; SPSS Inc., Chicago, USA). Student’s t-test was applied to compare the expression levels of PLAU and miRNAs between HNSCC and para-carcinoma tissues. Wilcoxon rank-sum test was performed to investigate the association between PLAU expression and the clinical parameters of patients with HNSCC. Univariate and multivariate Cox regression models with a hazard ratio were used to predict the prognostic ability of PLAU. *P-values* < 0.05 were considered to indicate a significant difference.

## Results

### Gene expression profile analysis and PLAU filtering

**Fig. 1:**
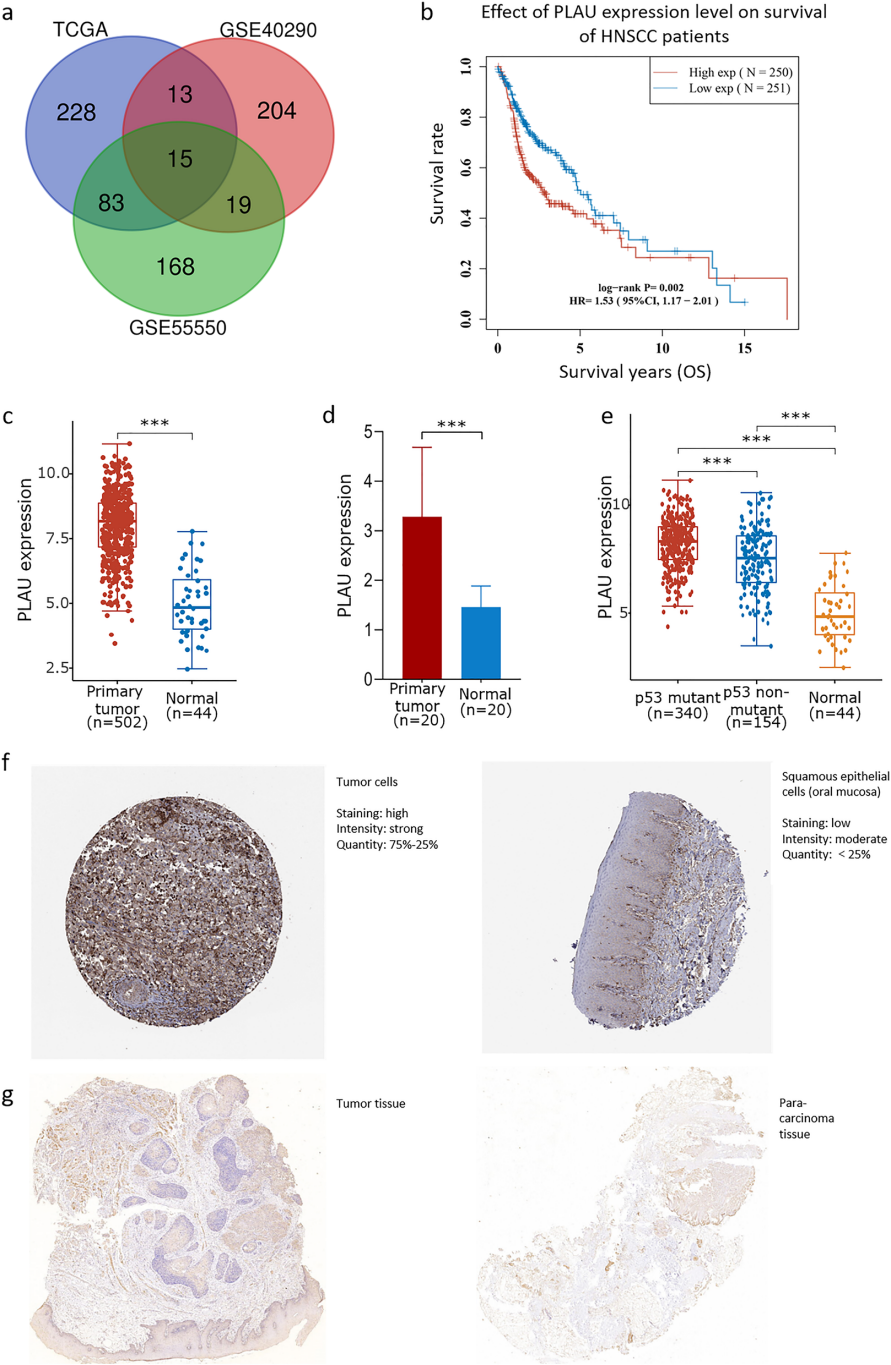
PLAU filtering and its expression in HNSCC. **a** Fifteen three-crossing and 115 two-crossing DEGs were filtered among three public datasets. **b** KM survival analysis showed that high expression of PLAU was associated with worse prognosis of patients with HNSCC. The mRNA level of PLAU was significantly higher in HNSCC compared to the normal para-carcinoma tissues from TCGA database (**c**) and our patients (**d**). The mRNA level of PLAU was especially higher in the p53 mutant group compared to the nonmutant group (**e**). The protein level of PLAU was much higher in HNSCC (left) than in the normal tissues of the oral mucosa (right) both from THPA (**f**) and our patients (**g**)

The adjusted *p* value was analyzed to correct for false positive results in TCGA or GEO. An adjusted *p* < 0.01 and log (Fold Change) > 2 or log (Fold Change) < − 2 were defined as the thresholds for screening. There were 285 and 251 DEGs between HNSCC and normal oropharyngeal or nasopharyngeal tissues in GSE55550 and GSE40290, respectively, and there were 339 DEGs between HNSCC and para-carcinoma tissues in TCGA. Two-crossing or three-crossing genes were discovered and visualized via Bioinformatics & Evolutionary Genomics (http://bioinformatics.psb.ugent.be/webtools/Venn/) among different datasets. A total of 15 three-crossing and 115 two-crossing DEGs (83 between TCGA and GSE55550, 13 between TCGA and GSE40290, and 19 between GSE55550 and GSE40290) were filtered (Fig. 1a and Additional file 1). Three DEGs were filtered by KM survival analysis (Additional file 2). We selected PLAU in this study for its lowest *p* value and highest hazard ratio (Fig. 1b).

### High expression of PLAU in HNSCC

PLAU was significantly upregulated in HNSCC tissues compared to normal para-carcinoma tissues from TCGA database (Fig. 1c) and our patients (Fig. 1d). Interestingly, when we divided HNSCCs according to the mutation status of p53, a curtail tumor suppressor gene that is extensively mutated in various human cancers, the PLAU was overexpressed in the p53 mutant group compared to the nonmutant group (Fig. 1e). Moreover, the protein expression level was significantly higher in HNSCC tissues than in the normal tissues of oral mucosa based on the THPA tool (Fig. 1f). Similar results were found in our patients (Fig. 1g). Moreover, as shown in Table 1, there were no significant associations between PLAU expression and the major clinical variables (such as tumor stage, lymph mode metastasis and clinical stage) or demographic variables (such as age, sex, and smoking status), indicating that a higher PLAU expression level contributed to a worse survival outcome, independent of other clinical parameters.

**Table 1:** Association between PLAU expression and clinical variables in patients with HNSCC

Clinical variable	Number	PLAU expression	*p-*value
Sex			
Male	368	7.96 ± 1.290	0.572
Female	134	8.05 ± 1.301	
Age (years)			
≥ 60	256	7.97 ± 1.280	0.748
< 60	221	7.98 ± 1.334	
Tumor stage			
T1	34	7.58 ± 1.342	0.088
T2	144	7.93 ± 1.296	
T3	133	7.93 ± 1.232	
T4	177	8.10 ± 1.277	
Lymph metastasis			
N0	241	7.94 ± 1.291	0.552
N1	81	8.10 ± 1.276	
N2	136	7.99 ± 1.261	
Clinical stage			
I	25	7.90 ± 1.166	0.726
II	81	7.93 ± 1.199	
III	90	7.99 ± 1.261	
IV	306	8.00 ± 1.339	
Smoking status			
Yes	381	8.00 ± 1.252	0.529
No	111	7.93 ± 1.380	

Complete data were unavailable in the TCGA database

^a^Tumor stage: T category of the TNM classification which described the primary tumor site and size

^b^Clinical stage: the TNM combinations were grouped into four less-detailed stages, including Stages I to Stage IV

### PLAU is an independent prognostic factor in HNSCC

The results of univariate Cox regression analysis revealed that the expression of PLAU and the clinical stage were important prognostic elements for patients with HNSCC (Table 2). To reduce the influence of other clinical parameters on the prognostic capacity of PLAU, multivariate Cox regression analysis was also applied, and the results showed that PLAU and clinical stage were independent prognostic factors (Table 3).

**Table 2:** Univariate Cox regression analysis of prognostic factors of HNSCC

Variable	OS		
	RR	95% CI	*p-*value
Sex (male/female)	0.774	(0.565, 1.062)	0.112
Age, years (≥ 60/< 60)	0.848	(0.630, 1.141)	0.275
Smoking status (yes/no)	0.705	(0.751, 1.528)	0.705
Tumor stage (T1/T2/T3/T4)	1.107	(0.946, 1.294)	0.205
Lymph metastasis (N0/N1/N2)	1.150	(0.973, 1.358)	0.101
Clinical stage (I/II/III/IV)	1.379	(1.149, 1.655)	0.001*
PLAU expression (high/low)	1.652	(1.228, 2.222)	0.001*

*OS* overall survival, *RR* relative risk, *CI* confidence interval

*p < 0.05

**Table 3:** Multivariate Cox regression analysis of prognostic factors of HNSCC

Variable	OS		
	RR	95% CI	*p-*value
Sex (male/female)	0.745	(0.536, 1.038)	0.082
Age, years (≥ 60/ < 60)	0.823	(0.607, 1.117)	0.211
Smoking status (yes/no)	1.065	(0.732, 1.549)	0.742
Tumor stage (T1/T2/T3/T4)	0.865	(0.710, 1.054)	0.150
Lymph metastasis (N0/N1/N2)	1.012	(0.839, 1.221)	0.901
Clinical stage (I/II/III/IV)	1.531	(1.194, 1.963)	0.001*
PLAU expression (high/low)	1.639	(1.216, 2.209)	0.001*

*OS* overall survival, *RR* relative risk, *CI* confidence interval

*p < 0.05

### Hypomethylation of PLAU in HNSCC

**Fig. 2:**
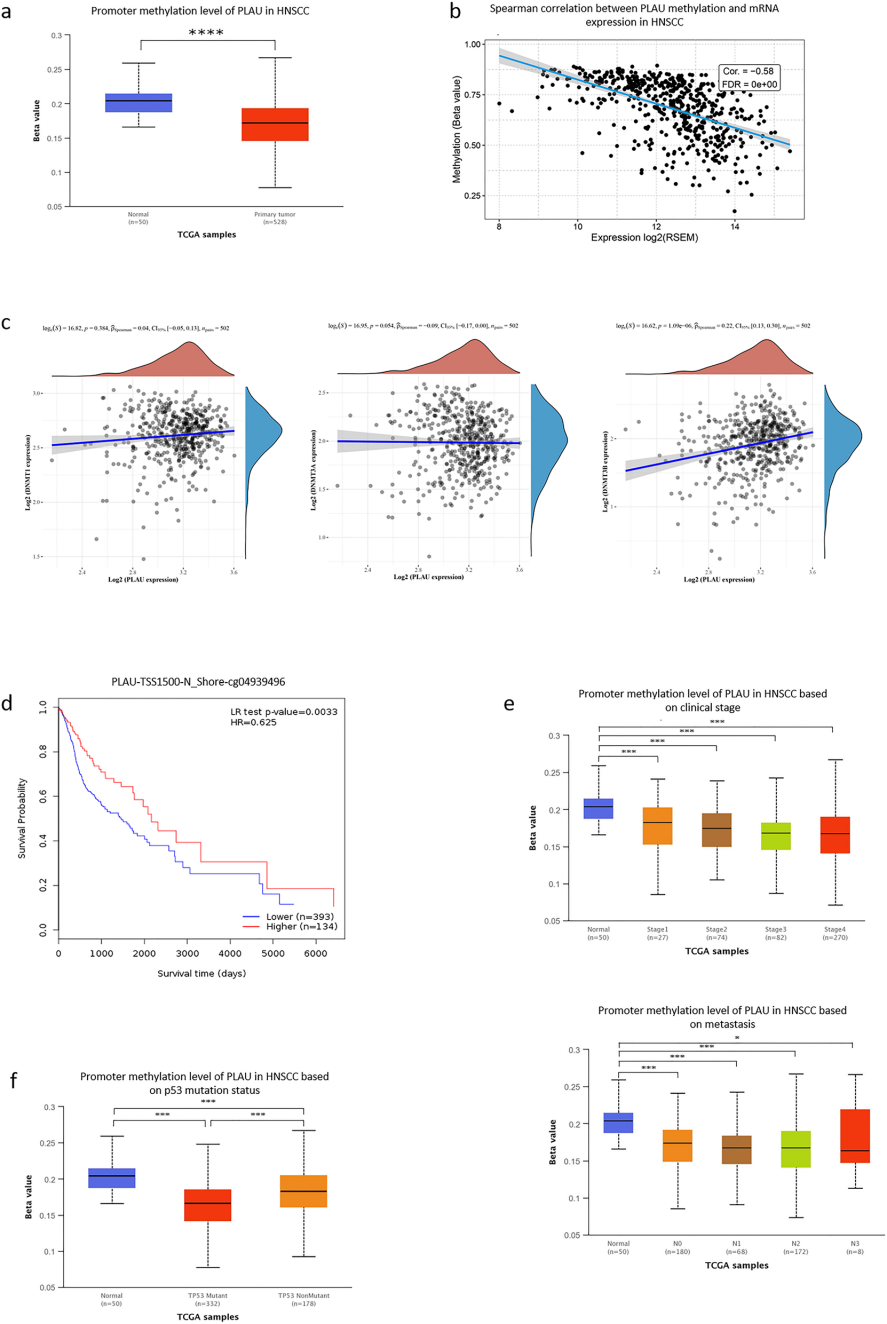
Hypomethylation of PLAU and its prognostic value in HNSCC. **a** PLAU was significantly hypomethylated in HNSCC tissues compared to the normal group. **b** PLAU methylation was negatively associated with PLAU expression. **c** No significant association was found between PLAU expression and DNMT1 (left) or DNMT3A (middle) levels. The positive correlation between PLAU expression and DNTM3B was significant (right). **d** Higher methylation of PLAU predicted a longer survival time compared to hypomethylation. **e** Lower methylation was correlated with higher clinical stage (above) and more aggressive metastasis (below). **f** PLAU methylation was lower in p53 mutant group than in the nonmutant group

To further clarify the mechanism underlying the overexpression of PLAU in HNSCC we investigated the methylation status via multiple tools. The analysis of UALCAN demonstrated that PLAU was significantly hypomethylated in HNSCC tissues compared to normal para-carcinoma tissues (Fig. 2a). Moreover, Spearman’s correlation analysis demonstrated that PLAU methylation was negatively associated with the mRNA expression level of PLAU (Fig. 2b). Besides, there was a significant positive correlation between the expression levels of PLAU and DNMT3B, while no significant association between PLAU expression and DNMT1 or DNMT3A was found (Fig. 2c). Based on the methylation sites around CpG islands from MethSurve, the only methylated site was found in N Shore, and was located in the promoter region of TSS1500.

### PLAU methylation was associated with prognosis of HNSCC

Survival analysis showed that higher methylation of PLAU predicted a longer survival time compared to hypomethylation (*p* = 0.003, Fig. 2d). The results from UALCAN provided a more comprehensive view of the critical role of PLAU methylation in clinical characteristics. Hypomethylation occurred in HNSCC, and lower methylation was correlated with higher clinical stage and more aggressive metastasis (Fig. 2e). Moreover, PLAU methylation was lower in p53 mutant samples than in nonmutant samples (Fig. 2f).

### PLAU interacted with multiple activated pathways and proteins

**Fig. 3:**
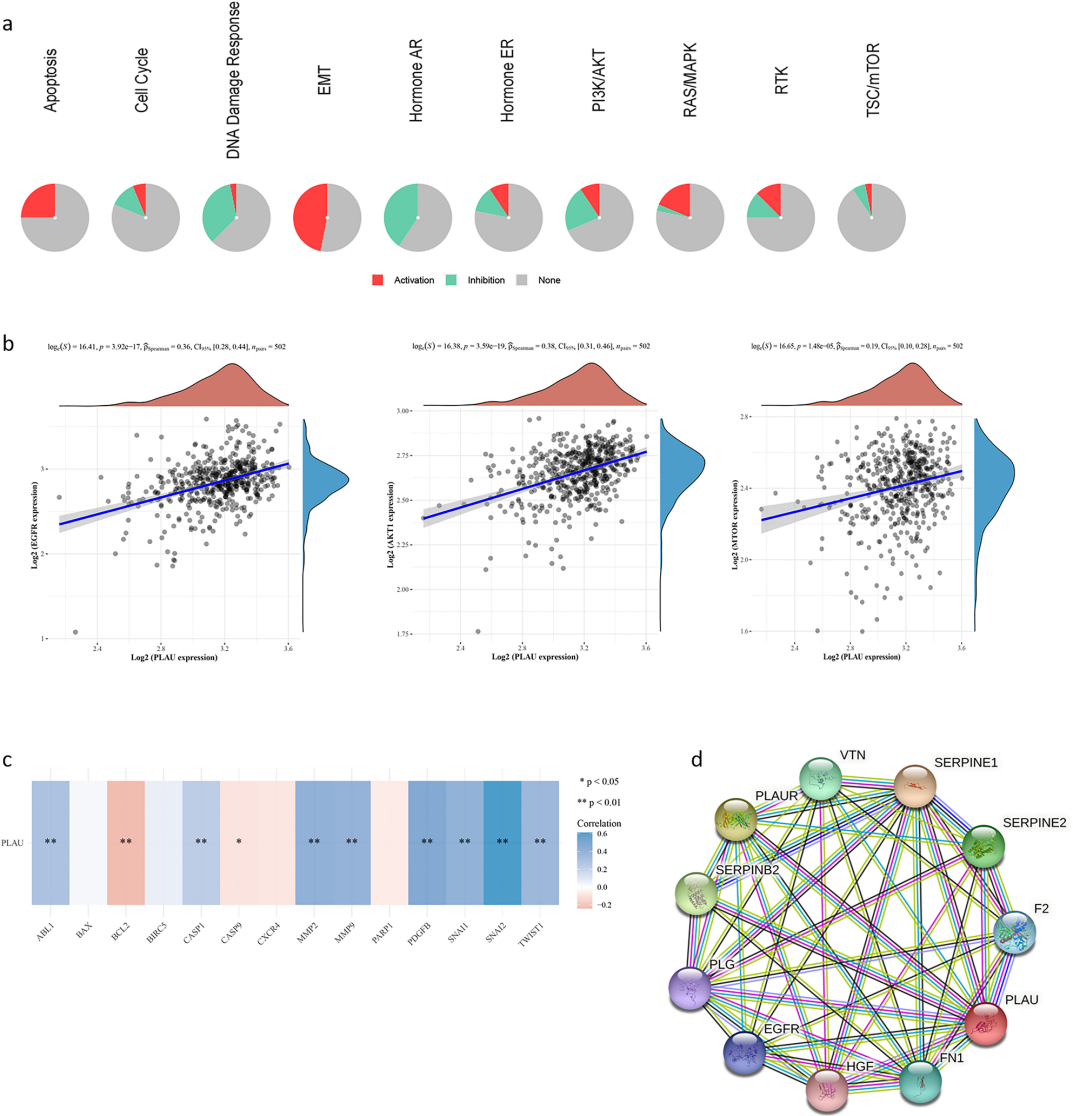
PLAU interacted with multiple activated pathways and proteins. **a** PLAU expression was related to multiple biological functions and signaling pathways, as known by GO and KEGG pathway analysis. **b** PLAU expression was positively correlated with EGFR (left), Akt1 (right) and mTOR (below). **c** The correlation between the mRNA expression levels of PALU and genes associated with apoptosis and the EMT pathway. **d** The PPI network of the top ten genes associated with PLAU

Analysis via GSCALite based on TCGA database revealed that PLAU expression was involved in multiple signaling pathways, including apoptosis, epithelial–mesenchymal transition (EMT), and Ras/MAPK (Fig. 3a). A valuable correlation was found between PLAU expression and some representative molecules, including EGFR, Akt1, and mTOR, in the corresponding pathways (Fig. 3b). The associations between PLAU and pivotal genes in apoptosis and EMT pathways based on TCGA database were also explored. The results revealed that BCL2 and Caspase9 were negatively correlated with PLAU, while others, such as ABL1, MMP2, and SNAI2, were positively correlated with PLAU (Fig. 3c). The PPI network showed that PLAU was correlated with multiple genes. The top ten related genes were selected, and the interaction network of these genes was visualized by STRING and Cytoscape software (Fig. 3d).

### Expression of PLAU-targeted miRNAs in HNSCC

**Fig. 4:**
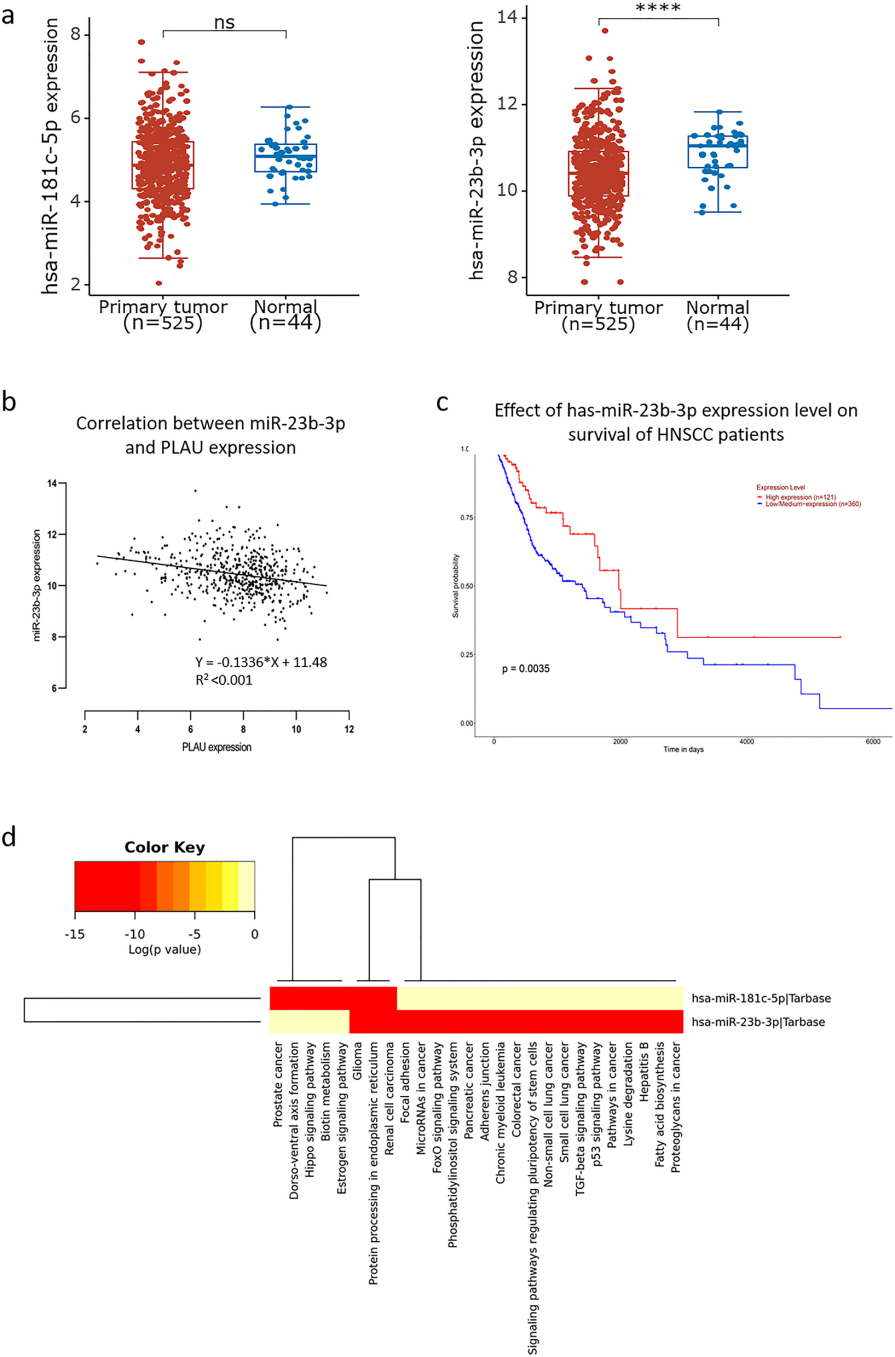
Expression and prognostic value of PLAU-targeted miRNAs in HNSCC. **a** MiR-181c-5p (*p* = 0.077, left) and miR-23b-3p (*p* < 0.001, right) were downregulated in HNSCC compared to normal tissues. **b** The expression level of miR-23b-3p was negatively correlated with PLAU expression. **c** Downregulation of miR-23b-3p predicted a worse prognosis in the 5-year survival of patients with HNSCC. **d** The biological functions and signaling pathways associated with miR-181c-5p and miR-23b-3p were predicted by GO and KEGG pathway analysis

PLAU-targeted miRNAs were predicted by various bioinformatics tools to further explore the mechanisms underlying PLAU upregulation in HNSCC. The overlapping miRNAs filtered from TargetScan, miRDB, and miCODE included miR-193a-3p, miR-193b-3p, miR-138-5p, miR-181a-5p, miR-181b-5p, miR-181c-5p, miR-181d-5p, miR-23a-3p, miR-23b-3p, and miR-23c. Among these miRNAs, miR-181c-5p (*p* = 0.077) and miR-23b-3p (*p* < 0.001) were downregulated in patients with HNSCC on the basis of TCGA database (Fig. 4a). The expression level of miR-23b-3p was negatively correlated with PLAU expression (y = − 0.1336x + 11.48, R^2^ < 0.001; Fig. 4b). Moreover, downregulation of miR-23b-3p predicted a worse prognosis in the 5-year survival of patients with HNSCC (Fig. 4c). GO analysis demonstrated the biological roles of PLAU-targeted miRNAs; the intersected miRNAs were enriched in the regulation of adherence, junctions and focal adhesion, among others. KEGG pathway analysis demonstrated that the overlapping genes were mainly focused on the p53 signaling pathway and the TGF-beta signaling pathway (Fig. 4d).

### PLAU is a potential diagnostic biomarker

**Fig. 5:**
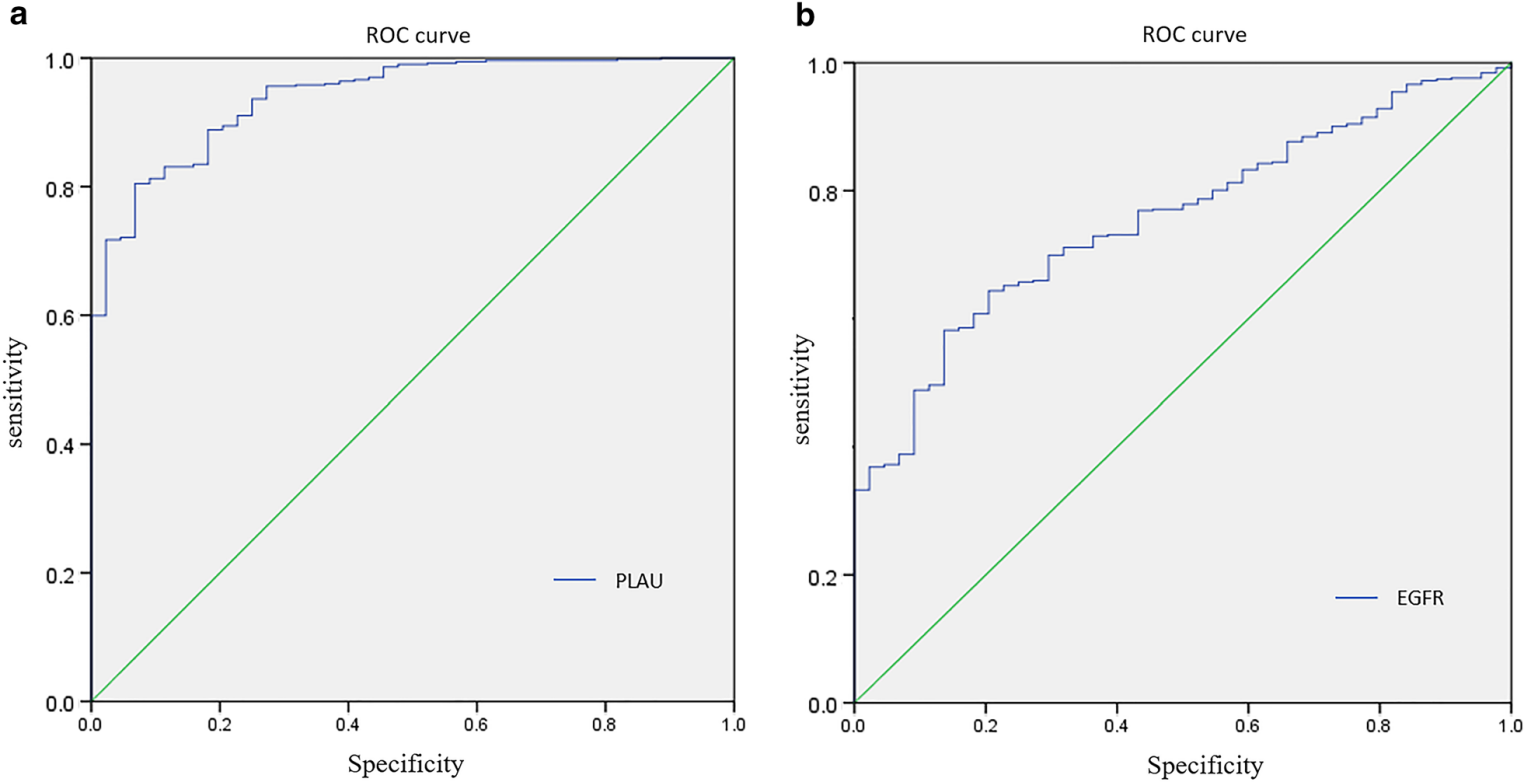
The diagnostic capability of PLAU in HNSCC was examined by ROC curve. The AUC of PLAU expression (**a**) was higher than that of EGFR (**b**)

The diagnostic capability of PLAU in the 5-year overall survival outcome of patients with HNSCC was examined by ROC curve. The area under the curve (AUC) of PLAU expression was 0.940 (Fig. 5a), higher than that of EGFR expression (AUC = 0.756, *p* < 0.001; Fig. 5b), which has been considered a biomarker in various malignant tumors. Taken together, these results indicate a strong diagnostic ability of PLAU for patients with HNSCC.

## Discussion

In this study, we filtered the DEGs between HNSCC and normal para-carcinoma tissues from three public datasets, and focused on PLAU given its prognostic significance via KM survival analysis. The results showed a close correlation between the upregulation of PLAU and poor outcomes of patients with HNSCC. Moreover, we used multiple bioinformatic tools to demonstrate that hypomethylation and PLAU-targeted miRNAs might play an important role in PLAU overexpression in HNSCC, leading to the promotion of tumorigenesis through the Ras/MAPK signaling pathway. To the best of our know, this is the first study to predict diagnostic and prognostic molecules in the perspective of methylation and miRNA regulation in HNSCC.

PLAU, also known as urokinase-type plasminogen activator (uPA), functions as a protease and belongs to the serine peptidase of S1 of Clan PA [23]. PLAU activates plasminogen via conversion to plasmin [24], which then aids in the migratory cascade through degradation of the surrounding extracellular matrix (ECM) constructs, activation of matrix metalloproteinases (MMPs), and release of growth factors. Plasmin serves as a feed-forward mechanism on the uPA-uPAR complex by activating pro-uPA to active uPA, resulting in the enhancement of cell migration and invasion [25]. Recently, researchers have focused on PLAU and its interactome in malignant tumors due to its role in proliferation, metastasis, and angiogenesis, and its aberrant expression in various cancers, suggesting its use as a diagnostic biomarker and therapeutic target [26–28]. It has been shown that in the PLAU or plasminogen deficient mice model, distal metastasis was decreased while no effect was found on tumor growth [29]. However, inhibition of PLAU expression has been shown to prevent the migration and invasion of cervical cancer cells through the downregulation of MMP2 [30].

Growing evidence suggests that aberrant DNA methylation, an epigenetic modification, is closely associated with the occurrence, development, and prognosis of various cancers [31–34]. Two patterns of DNA methylation are related to tumorigenesis: promoter domain CpG island hypermethylation and genome-wide hypomethylation [35]. The genes affected by DNA methylation are associated with a cluster of cellular pathways, including cell proliferation, apoptosis and invasion [36, 37], and are more widely found in almost all tumors than other types of DNA abnormalities, such as mutations, abnormal gene amplification and chromosomal abnormalities [38]. In our study, we evaluated PLAU methylation by multiple bioinformatics tools. Consistent results showed that PLAU was hypomethylated in HNSCC tissues, which provided us with an understanding of the PLAU overexpression in HNSCC. In agreement accordance with our findings, a previous study reported that DNA methylation has a crucial influence on gene expression [39]. Additionally, inspired by the positive association between PLAU hypomethylation and the prognosis of patients with HNSCC, as well as the correlations between PLAU methylation and some clinical variables (tumor stages and metastasis), this epigenetic aberrance may increase the risk of HNSCC-related death. Therefore, methylation-associated activation of PLAU might be considered a target in future research. Besides, although we have found positive correlations between the mRNA levels of PLAU and DNMT3B, ChIP-seq should be performed to establish whether hypomethylation of PLAU in promoters is caused by methyltransferases.

To further explore whether additional mechanisms underlie the upregulation of PLAU in HNSCC, multiple online tools were executed to predict PLAU-targeted miRNAs. Among the overlapping miRNAs, miR-23b-3p was downregulated in HNSCC and negatively correlated with PLAU expression, which provided us with another explanation for the overexpression of PLAU. Besides, the level of miR-23b-3p was positively correlated with the survival of patients with HNSCC, suggesting that miR-23b-3p/PLAU is a prognostic biomarker in HNSCC. Hu et al. [40] found that miR-23b-3p could prevent the expression of ATG12, attenuating the chemo-induced autophagy and chemoresistance in gastric cancer cells. Additionally, miR-23b-3p has been found to be significantly downregulated in the exosomes and serum collected from patients with prostate cancer, which promoted cell proliferation and invasion via EMT process [41]. Gabriela et al. [42] reported that miR-23b-3p acted as a tumor suppressor in cervical cancer by directly repressing c-Met expression and overexpression of miR-23b-3p reduced the proliferation, migration, and invasion of cervical cancer cells. Although previous studies have revealed the protective role of miR-23b-3p in the occurrence, development, and prognosis of various malignant tumors, few have focused on miR-23b-3p in the context of HNSCC. To the best of our known, this is the first study to reveal the targeted relationship of miR-23b-3p and PLAU, and evaluate their roles in the prognosis of HNSCC.

Our study demonstrated that the Ras/MAPK pathway might affect PLAU overexpression in HNSCC. A previous study demonstrated that PLAU activates the MAPK signaling pathway and focal adhesion kinase systems [43]. Indeed, nearly all current small-molecule inhibitors targeting Ras/MAPK pathway components function to inhibit their kinase activity, which is critical for the phosphorylation and activation of their downstream effectors [44]. Pawel et al. [45] showed that protein lysine methylation controlled the phosphorylation status of a key component of the Ras/MAPK pathway to enable oncogenic KRAS in cancer progression, providing further understanding of the Ras/MAPK pathway in gene methylation.

In this study, a significant association between PLAU expression and mutated p53 was shown, suggesting that PLAU might be involved in the initiation and development of HNSCC. Besides, the expression levels of EGFR, Akt1 and mTOR were positively correlated with PLAU expression. It is known that the Akt/mTOR signaling route is also activated in concert with the Ras/MAPK pathway downstream of EGFR in HNSCC [46]. Additionally, mTOR inhibitors have been shown to induce apoptosis and wild-type p53 expression in two separate in vivo models of HNSCC [47], while the inhibition of Akt reversed the direct suppression of p53 by transcriptional and post-translational mechanisms. Therefore, therapies targeting Akt/mTOR may be doubly effective due to their capacity to limit both PI3K/Akt/mTOR-mediated survival and growth, and intensify p53-mediated apoptosis.

Despite the merits of this study, the limitations should be addressed. First, the analyses were conducted on the basis of public datasets via multiple bioinformatic tools, which could provide a new understanding for further research. Therefore, our findings need to be confirmed in vitro and vivo experiments. Besides, the platform applied in different cohorts is not the same, which may bring bias to the data analysis and difficulties for the deep integrated analysis. Second, we also evaluated PLAU as a prognostic factor separately for the three main sites of head and neck (oral cavity, pharynx and larynx). Third, KM survival analysis showed that patients with low expression levels of PLAU lived longer compared to those with higher PLAU expression in pharyngeal (*p* = 0.001) and oral (*p* = 0.02) squamous cell carcinoma groups. However, no significant differences were found in OS between PLAU^high^ and PLAU^low^ groups in patients with laryngeal squamous cell carcinoma (Additional file 3). This may be due to the relatively small sample size when we divided HNSCCs into three subgroups. Therefore, the results of our study must be applied with caution in patients with laryngeal squamous cell carcinoma, and a prospective cohort study will be performed to verify the prognostic role of PLAU in HNSCC.

In summary, we evaluated the diagnostic and prognostic capacity of PLAU based on TCGA database in HNSCC and demonstrated that hypomethylation and downregulation of miR-23b-3p were strongly associated with overexpression of PLAU via s Ras/MAPK and Akt/mTOR signaling pathways. Therefore, PLAU may be considered an independent biomarker for diagnosis and prognosis in HNSCC.

## Supplementary Information

**Additional file 1. **Overlapping genes among three public datasets.

**Additional file 2. **Differentially expressed genes with significant prognostic capacity in HNSCC.

**Additional file 3.** Effect of PLAU expression levels on survival of the patients with laryngeal, oral, and pharyngeal squamous cell carcinoma.

**Additional file 4.** PLAU expression levels in HNSCC patients with different HPV status.

## Data Availability

The datasets used and/or analysed during the current study are available from the corresponding author on reasonable request.

## Abbreviations

HNSCCs: Head and neck squamous cell carcinomas
TNM: Regional lymph nodes, metastasis
PLAU: Plasminogen activator urokinase
ROC: Receiver operation characteristic
GEO: Gene Expression Omnibus
TCGA: The Cancer Genome Atlas
DEGs: Differentially expressed genes
KM: Kaplan–Meier
THPA: The Human Protein Atlas
OS: Overall survival
PPI: Protein–protein interaction
KEGG: Kyoto Encyclopedia of Genes and Genomes
GO: Gene Ontology
EGFR: Epidermal growth factor receptor
EMT: Epithelial–mesenchymal transition
AUC: Area under the curve
uPA: Urokinase-type plasminogen activator
ECM: Extracellular matrix
