# Supplementary material for: Hypomethylation and downregulation of miR-23b-3p are associated with upregulated PLAU: a diagnostic and prognostic biomarker in head and neck squamous cell carcinoma
Source: Cancer Cell Int. 2021 Oct 26;21:564. doi: 10.1186/s12935-021-02251-w (PMC8549381; doi:10.1186/s12935-021-02251-w)
Supplement: Supplementary file 2 — Additional file 2. Differentially expressed genes with significant prognostic capacity in HNSCC. [file 12935_2021_2251_MOESM2_ESM.docx]

**Additional file 2** The Differentially expressed genes with significant prognostic capacity in HNSCC

| Genes | HR | Low 95% CI | Hight 95% CI | *p-*value |
| --- | --- | --- | --- | --- |
| FN1 | 1.318 | 1.006 | 1.726 | 0.045 |
| PLAU | 1.533 | 1.170 | 1.170 | 0.002 |
| FAM3D | 0.750 | 0.573 | 0.573 | 0.036 |

HR: Hazard ratio, CI: Confidence interval.
