# Supplementary figures and images for: Hypomethylation and downregulation of miR-23b-3p are associated with upregulated PLAU: a diagnostic and prognostic biomarker in head and neck squamous cell carcinoma
Source: Cancer Cell Int. 2021 Oct 26;21:564. doi: 10.1186/s12935-021-02251-w (PMC8549381; doi:10.1186/s12935-021-02251-w)

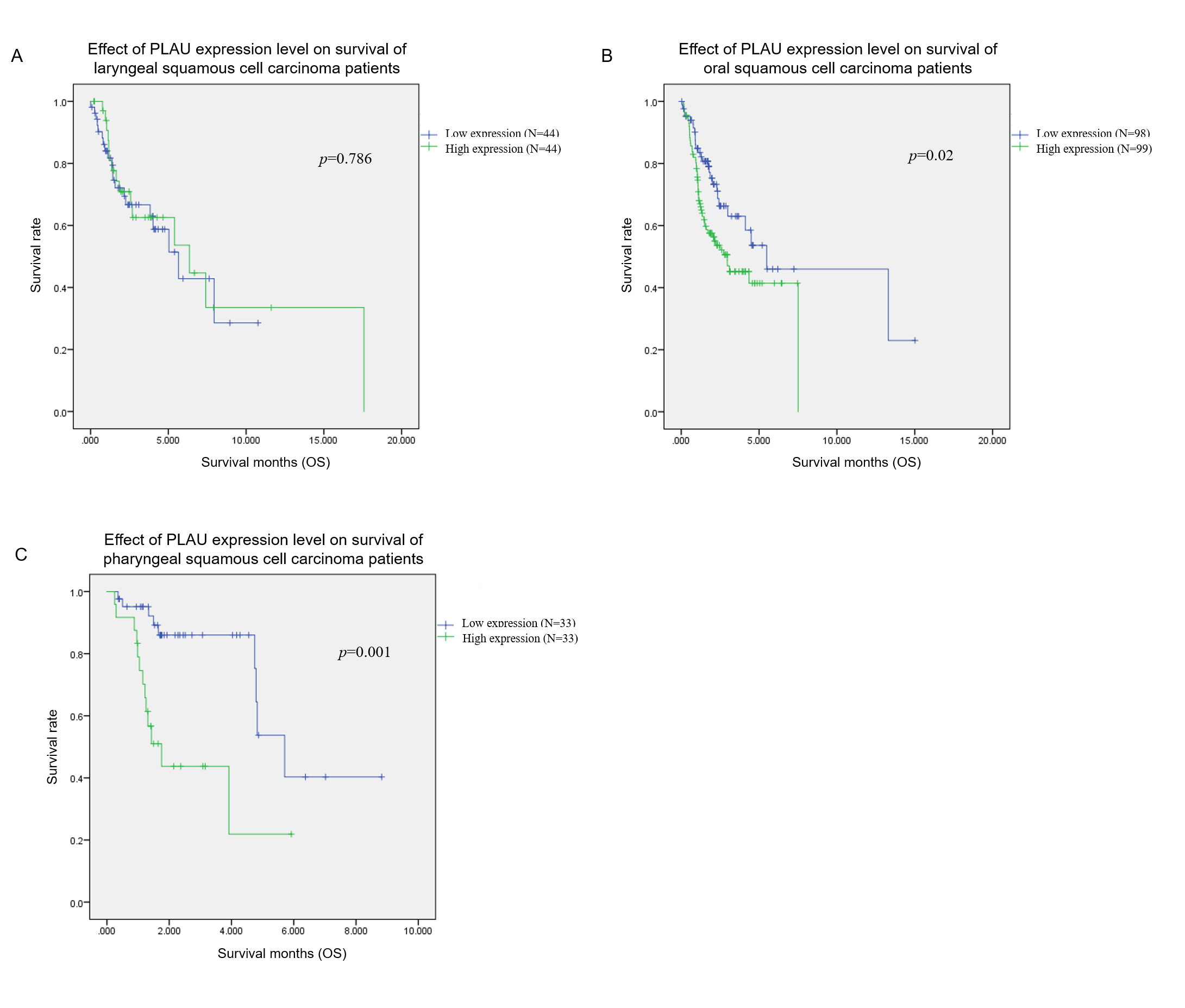

Supplement: Supplementary file 3 — Additional file 3. Effect of PLAU expression levels on survival of the patients with laryngeal, oral, and pharyngeal squamous cell carcinoma. [file 12935_2021_2251_MOESM3_ESM.tif]

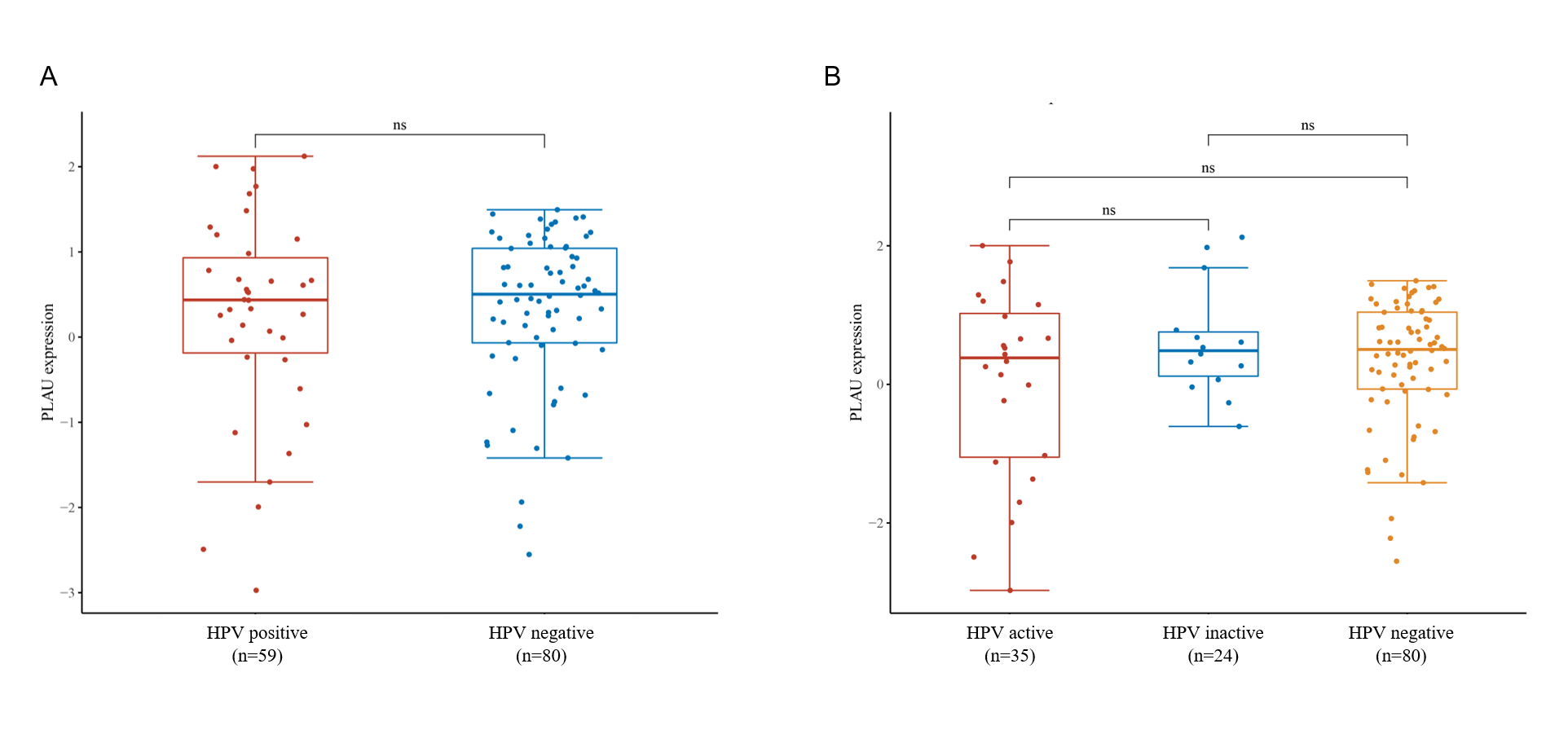

Supplement: Supplementary file 4 — Additional file 4. PLAU expression levels in HNSCC patients with different HPV status. [file 12935_2021_2251_MOESM4_ESM.tif]
